# Adverse Interaction between Capecitabine and Warfarin Resulting in Altered Coagulation Parameters: A Review of the Literature Starting from a Case Report

**DOI:** 10.1155/2010/426804

**Published:** 2010-07-06

**Authors:** Giovanni Giunta

**Affiliations:** ASL 10, “Veneto Orientale”, Hospice Oncologico, Portogruaro, Borgo San Gottardo 44, Venice, Italy

## Abstract

Capecitabine is an orally active prodrug of fluorouracil and is extensively used as an antineoplastic agent. It is converted to 5-Fluorouracil in the liver and tumor tissues. Warfarin is an anticoagulant agent for preventing and treating venous and arterial thrombosis and embolism and is metabolized by cytochrome P450 isoenzymes in the liver. Preclinical in vitro studies using human liver microsomes report no inhibitory effects between capecitabine and substrates of cytochrome P. However, the concomitant administration of capecitabine and warfarin resulted in INR elevation in the cases previously reported in the literature. The exact mechanism of this interaction is unknown but may be related to downregulation of cytochrome P450 2C9 by capecitabine or its metabolites. We report on the possible adverse interaction between capecitabine and warfarin in a patient with metastatic breast cancer and critically review the existing literature on this topic. Physicians should be aware of adverse reactions arising from the combined use of capecitabine and warfarin. In the light of the current data, INR levels should be closely monitored in patients using these drugs together.

## 1. Introduction

Capecitabine is an orally administered prodrug of 5-fluorouracil (5-FU) that mimics continuous infusion of 5-FU and generates 5-FU preferentially at the tumor site. In vitro human liver microsome assays have not suggested any significant potential for interaction between capecitabine or its metabolites and drugs which are substrates for the cytochrome P450 (CYP) isoenzymes 1A2, 2A6, 2C9, 2C19, 2D6, 2E1, or 3A4 (http://www.rocheusa.com/products/ xeloda/pi.pdf), which would include anticoagulant coumarin derivates. By contrast, several case reports in the literature have provided clinical evidence for a possible interaction between capecitabine and coumarin derivatives [1–5] A retrospective study [6] investigated coagulation abnormalities in patients receiving concomitant capecitabine and warfarin, finding an INR >3 in one third of the patients on warfarin versus 4% for those not on warfarin, with a higher incidence of bleeding in the first group.

## 2. Case Report

A 59-year-old female with metastatic breast cancer (sites of metastases were bone and lung without liver involvement) was receiving chronic minidose of warfarin therapy (1 mg/day) for prophylaxis against catheter-associated thrombosis. Her prior chemotherapy included an adriamycin-based regimen given until disease progression. She had an hormone-receptor negative and high grade tumour, but she was in good general conditions, with an ECOG performance status of 0 and a life expectancy of more than 3 months. The laboratory tests were within the normal limits and her cardiac function was normal. The international normalized ratio (INR) level was monitored every week and was, prior to initiating capecitabine, always under the value of 2. In may 2005 she received the first course of chemotherapy using capecitabine 1000 mg/m^2^orally twice daily for a period of 14 days, followed by a rest period of 7 days and intravenous (iv) docetaxel 75 mg/m^2^ on day 1 and then every 3 weeks. Dexamethasone 20 mg was administered orally the night before and the day of chemotherapy and then at dose of 8 mg twice daily for a period of 2 days after docetaxel administration. Ondansetron, 8 mg twice daily, was given as an antiemetic on the day of docetaxel treatment. Prior to the docetaxel administration, ranitidine 50 mg was infused iv over 15 min, followed by 10 mg chlorpheniramine. Twenty-one days after initiating capecitabine, the patient's INR was markedly elevated (8, 87). Neither symptoms nor signs of bleeding were detected. The patient did not report any changes in dietary habits or physical activities, use of other drugs, alcohol or smoke, presence of fever or hyperthyroidism that could explain her condition. Twenty-four hours after the iv administration of Vitamin K 10 mg the INR value had dropped to 1.19. The warfarin was withheld and the patient did not develop any complications. The patient was treated with low molecular weight heparin subcutaneusly and the chemotherapy regimen was restarted without alterations of the coagulation parameters.

## 3. Review of the Literature and Discussion

Warfarin exerts its anticoagulant effect by interfering with the hepatic synthesis of vitamin K-dependent clotting factors II, VII, IX, and X and proteins C and S. The pharmacokinetic (PK) and pharmacodynamic (PD) properties of warfarin, as well as its narrow therapeutic index make it particularly susceptible to interactions with other drugs, and increased prothrombin time monitoring and warfarin dosing adjustments are required to maintain safe and effective anticoagulation. Minidose (1 mg/d) warfarin is often utilized in clinical practice to prevent venous thrombosis in cancer patients [7] as well as for thrombotic prophylaxis for central venous catheters [8]. Minidose warfarin is considered safe and monitoring of the prothrombin time is not needed. Capecitabine is indicated, alone or in combination with other drugs, for the treatment of patients with metastatic breast cancer. Capecitabine is also approved for use in metastatic colorectal cancer. Capecitabine is a fluoropyrimidine carbamate which is enzymatically converted to 5-FU *in vivo* [9] preferentially at the tumour site. Capecitabine is readily absorbed by the gastrointestinal tract. In the liver, a 60 kDa carboxylesterase hydrolyzes much of the compound to 5′-deoxy-5-fluorocytidine (5′-DFCR). Cytidinedeaminase, an enzyme found in most tissues, including tumors, subsequently converts 5′-DFCR to 5′-deoxy-5-fluorouridine (5′-DFUR). The enzyme, thymidine phosphorylase (dThdPase), then hydrolyzes 5′-DFUR to the active drug, 5-FU. Many tissues throughout the body express thymidine phosphorylase. Some human carcinomas express this enzyme in higher concentrations than surrounding normal tissues. A recent study suggests that patients with high levels of intratumoral Thymidine phosphorylase expression are the ideal candidates for capecitabine-based chemotherapy [10]. Plasma protein binding of capecitabine and its metabolites is less than 60% and is not concentration dependent. Capecitabine and its metabolites are predominantly excreted in urine, fecal excretion is minimal. The major metabolite excreted in urine is the $\tilde{\alpha }$fluoro-*β*-alanine [FBAL] which represents 57% of the administered dose. About 3% of the administered dose is excreted in urine as unmodified drug. Drugs may interact with warfarin through PD or PK mechanisms. PD mechanisms for drug interactions with warfarin are synergism (impaired hemostasis, reduced clotting factor synthesis), competitive antagonism (vitamin K), and altered physiologic control loop for vitamin K metabolism (hereditary resistance). PK mechanisms for drug interactions with warfarin are mainly enzyme induction, enzyme inhibition, and reduced plasma protein binding. It is important to mention that some drugs may interact by more than one mechanism. The mechanism of action for the interaction between warfarin and capecitabine is not clear, but may be related to downregulation of CYP2C9 by capecitabine or its metabolites. Moreover, capecitabine has a low potential for PK interactions related to plasma protein binding. An important study was conducted in two clinical centers in Scotland and United Kingdom with the primary objective to investigate in detail the effect of capecitabine on the pharmacokinetics and pharmacodynamics of warfarin in a clinical setting [11]. In this study, 3 women and 3 men suffering from metastatic breast or colorectal cancer were enrolled. This study assessed the influence of capecitabine on the PK (Plasma concentrations) and PD (INR and Factor VII) of warfarin. Plasma concentrations of capecitabine and its metabolites were not influenced by warfarin. None of the patients had concomitant treatments which were CYP2C9 inducers or inhibitors. This study clearly showed a marked INR elevation in the presence of combined capecitabine and warfarin treatment. Mean baseline factor VII levels dropped while on capecitabine therapy, potentially contributing to the observed PD interaction, though this effect did not reach statistical significance.

Warfarin consists of a pair of enantiomers that are extensively and differently metabolized by human CYP isoenzymes [12]. Descriptive and comparative statistics of the PK of the two enantiomers of warfarin (S- and R-Warfarin) on days 1 and 61 revealed that higher exposure to *S*-warfarin was obtained with concomitant administration of warfarin plus capecitabine compared with warfarin alone. Coadministration of capecitabine increased the area under the plasma concentration time curve from 0 to infinity (AUC_0−*∞*_) of *S*-warfarin by 57% and its elimination half-life (*t*_1/2_) by 51%. Apparent clearance (CL/F) was decreased by 36%. Time to reach maximum plasma concentration (*t*_max_) was doubled, but the maximum plasma concentration (*C*_max_) of *S*-warfarin was unaffected. In contrast, comparison of the PK parameters of *R*-warfarin on days 1 and 61 revealed only minor differences. *R*-warfarin is primarily metabolized by CYP1A2 and CYP3A4, while *S*-warfarin, the more active enantiomer, is metabolized almost exclusively by CYP2C9 [13, 14]. These data suggest that in humans capecitabine or its metabolites may be interacting with CYP2C9, but that they are less likely to be having any major effect on CYP3A4 or CYP1A2. Clinically relevant thromboses are relatively common within the oncology setting, in association with either the underlying malignancy or its treatment [15]. In a more recent study, patients with malignancy, compared with nonmalignant patients, had a statistically significant increase in overall incidence of recurrent thromboembolic events (27.1 v 9.0, respectively, per 100 patient-years) as well as bleeding (13.3 v 2.1, respe., per 100 patient-years) [16]. In both groups of patients, the incidence of thromboembolic events was lower when the INR was above 2.0 compared with below 2.0. Therapeutic or prophylactic anticoagulation with coumarin derivatives is therefore a frequent backdrop to anticancer intervention with chemotherapeutic agents [17]. The capecitabine-warfarin interaction is clinically significant, requiring a black box warning in the package insert. The underlying mechanism of this interaction seems to be the enzyme downregulation, rather than direct enzyme inhibition [1]. In addition, several case series documenting interactions between warfarin and 5-FU, suggest the potential for a more general fluoropyrimidine-warfarin class effect [18–20]. The INR elevation may occur when 5-FU is given along with either low and full-doses of warfarin [18, 19, 21, 22]. In a study [21] the incidence of INR elevation was observed in 38 of 94 patients (40%) treated with Folfox regimen (5-FU, Oxaliplatin, leucovorin) and concomitant minidose of warfarin. Interestingly, in this study all patients with INR abnormalities had normal platelet counts and none had grade II–IV hepatic toxicity and INR elevations were observed more often in patients treated with the full dose of Folfox. Since the final step of the activation cascade of capecitabine generates 5-FU, it is reasonable to speculate that the noted clinical interaction between 5-FU or capecitabine and warfarin may be occurring through similar mechanisms. There is no direct evidence to confirm this, but it is worthy of remark that in clinical cases a long time delay has been reported before evidence of interaction between capecitabine and warfarin. Similar delays in the onset of clinically relevant interactions have been noted for 5-FU, and 5-FU administration does modulate the constitutive expression of certain CYP isoenzymes in a whole rat model [23]. Treatment with 5-FU produced a significant decrease in the total serum clearance value of *S*-enantiomer of warfarin in rats [24]. A direct PD interaction between capecitabine or 5-FU and warfarin, involving vitamin K and/or levels of active clotting factors could maybe have contributed to the observed INR changes. 5-FU, and by extension, capecitabine-induced mucositis within the gastrointestinal tract may limit the intake and/or the absorption of vitamin K, increasing the sensitivity of patients to vitamin K antagonists, such as warfarin. Nevertheless, an effect on vitamin K intake/absorption is unlikely to be a major contributing factor, considering that in the literature no clear links between the severity of fluoropyrimidine toxicity in the gastrointestinal tract and the degree of INR elevation in the presence of warfarin have been noted. A retrospective study of 77 patients [25] who received capecitabine was performed to analyze coagulation abnormalities with or without warfarin. Thirteen patients (11 on warfarin) had an INR >3 (range, 3.23–11.5), resulting in a probability of an INR >3 of 32% in the warfarin group versus 4% for those not on warfarin. The incidence of bleeding at 130 days of treatment with capecitabine was 18% with warfarin versus 2% without. Bleeding episodes were not significantly different between patients with or without liver involvement (4 of 40 episodes versus 3 of 37 episodes, resp.; *P* = .12). Patients with an INR >3 were evenly distributed between those with or without liver involvement (6 of 40 patients versus 7 of 37 patients, resp.). No INR increases persisted after discontinuation of capecitabine. Recently, the first case of markedly increased anticoagulant activity of warfarin when used in combination with doxifluridine has been reported [26], clearly indicating that the anticoagulant activity of warfarin was markedly increased by the concomitant use of doxifluridine as well as capecitabine or 5-Fu. Larger studies with capecitabine and other fluoropyrimidines will be required in the future to fully investigate the exact mechanism by which the interaction occurs and to understand the extent to which direct effects on clotting factor activity levels contribute toward INR changes in any noted fluoropyrimidine-warfarin interaction.

## 4. Conclusions

Several case reports in the literature have provided clinical evidence that fluorouracil-based chemotherapy regimens may potentiate the effects of coumarin derivatives. Possible interactions between warfarin and capecitabine have been reported and may occur at any time. In addition, a study [11] demonstrates a clear PD interaction between capecitabine and *S*-warfarin, resulting in exaggerated anticoagulant activity. This interaction is probably due to an inhibition of cytochrome P450 2C9 by capecitabine and/or its metabolites. These events occurred in patients with and without liver metastases. It is reasonable to speculate that the interactions between fluoruracil or capecitabine and warfarin may be occurring through similar mechanisms. These events occur within several days and up to several months after initiating fluoropyrimidine therapy. These data provide an obvious justification for the advice that patients receiving concomitant oral warfarin anticoagulant therapy and a fluoropyrimidine should have their INR monitored closely throughout treatment and for 1 month after and their warfarin dose adjusted accordingly. This is particularly relevant to patients receiving long-term treatment whose warfarin dose may have been considered to be stable before starting a fluoropyrimidine. In the event of severe or uncontrollable interactions, if anticoagulation is still indicated, noncoumarin-based approaches, for example, with low molecular weight heparin, should be considered as an alternative.
